# Transposable elements in *Drosophila*

**DOI:** 10.1186/s13100-020-00213-z

**Published:** 2020-07-03

**Authors:** Vincent Mérel, Matthieu Boulesteix, Marie Fablet, Cristina Vieira

**Affiliations:** Université de Lyon, Université Lyon 1, CNRS, Laboratoire de Biométrie et Biologie Evolutive UMR 5558, F-69622 Villeurbanne, France

**Keywords:** Population genomics, *Drosophila*, intra and interspecific TE diversity, epigenetics

## Abstract

*Drosophila* has been studied as a biological model for many years and many discoveries in biology rely on this species. Research on transposable elements (TEs) is not an exception. *Drosophila* has contributed significantly to our knowledge on the mechanisms of transposition and their regulation, but above all, it was one of the first organisms on which genetic and genomic studies of populations were done. In this review article, in a very broad way, we will approach the TEs of *Drosophila* with a historical hindsight as well as recent discoveries in the field.

## Background

### A few words about Transposable Elements

Transposable elements (TEs) are selfish genetic elements that are able to multiply in a genome by copying themselves to other locations. This particular property allows them to persist and multiply in populations without the need of providing any advantage to the host [1–3]. Discovered in maize in the late 1940’s by Barbara McClintock, they were understudied for decades [4, 5]. With the advent of molecular biology, notably their use for genetic engineering, an enormous amount of work has been done on TEs. The first sequencing projects stimulated the interest in these sequences, as they underscored their ubiquitous character. Indeed, TEs are found in virtually all eukaryotic species investigated so far [6–9]. They may represent up to 80% of a genome, as in Maize [10]. Additionally, one may expect these large elements, up to 20 kb, possessing coding sequences, regulatory sequences, and a unique epigenetic profile, to produce large-effect mutations [11, 12]. Actually, TEs have been shown to profoundly impact not only genomes, from chromosomal rearrangements to genome size, but also individuals, from deleterious to adaptive effects. Like many other research topics in biology, research on TEs owes much to *Drosophila*.

### A few words about *Drosophila*

The *Drosophila* genus is estimated to include several thousand species [13] sharing their most recent common ancestor ~25-40 My ago [14]. So far, ~1500 drosophilid species have been described. The most extensively studied *Drosophila* species is, by far, *Drosophila melanogaster*. Originating from Sub-Saharan Africa, it has colonized all continents, except for Antarctica, as a human commensal [15, 16]. During the last 15,000-20,000 year, it expanded its range to Europe and Asia and was only recently introduced to Australia and the Americas (~200 years ago) [17]. *D. melanogaster* is raised in the lab since the beginning of the XXth century [16, 18]. Easy to maintain and having a short generation time, this species has been extensively studied since then. Nowadays, a search for the terms “*Drosophila*” and “*melanogaster*” on *pubmed* returns approximately 55,000 references, with more than 2000 published in 2018.

A great number of genetic tools, such as genetic transformation vectors using TEs, and the P-element in particular [19], the GAL4/UAS system to study gene expression, or more recently, the CRISPR/Cas9 system for site-specific genome engineering, are available for *Drosophila* species (see [18] for review). In addition to genetic tools, genome sequencing is relatively easy in this genus. Due to their relatively small size, *Drosophila* genomes can be sequenced at relatively low cost [20]. *D. melanogaster* genome was among the first eukaryotic genomes sequenced, and is arguably the best annotated genome so far. A lot of sequencing data are available in the *Drosophila* genus. The genome of at least 46 species were sequenced and assembled [21]. In addition, in *D. melanogaster*, several studies aimed at sequencing either individuals or populations (PoolSeq) [22–29]. This sequencing effort benefited largely from diverse consortia. One of the first, and probably one of the best-known**,** the *Drosophila melanogaster Genetic Reference Panel (*DGRP) consortium made available the genomic sequence of more than two hundred inbred lines from an American population [22, 24]. At a broader geographical scale, the global diversity lines consortium sequenced a panel of 84 worldwide strains [29]. We also should mention the European *Drosophila* Population Genomics Consortium (*DrosEU*) which recently produced PoolSeq data fom 48 European population samples [28]. Nowadays, more than 1,121 individual Drosophila genomes are available [30], as well as pooled genomes from 30 localities in Europe and 23 in North America. For some individual genomes of the DGRP, data about gene expression and various phenotypic traits are also available [22, 31–34]. DGRP lines and a large variety of mutants and natural strains of *D. melanogaster,* collected from all over the world at different times, are currently maintained and available for researchers [35]. In addition, more than 250 species are accessible [36]. From an ecological/genomics perspective, *Drosophila* species offer a unique opportunity to perform comparative studies. For instance, the pair *D. melanogaster*/*D. simulans*, with a short time of divergence (around 1.5 My), share a common geographical range, as both are cosmopolitan species, but have very different ecologies, the former being close to human habitats and the second being found only in forest environments [14, 37]. Other *Drosophila species*, such as *D. suzukii*, are classified as invasive species, and represent an opportunity to study the genomic determinants of the invasive process. A last example that we can cite is the use of *Drosophila* species as models for speciation studies. This has been done extensively using the species close to *D. melanogaster* (*D. simulans*, *D. sechellia* and *D. mauritiana*) [38–40] and species from the *repleta* group (*D. mojavensis* and *D. arizonae* [41–44]; *D. buzzatii* and *D. koepferae* [45–47].

### A few words about Transposable Elements & *Drosophila*

*Drosophila* has been used as a model to study TEs for more than forty years now. The activity of the then-called “mobile dispersed genes” was already studied at the beginning of the 80’s [48, 49]. Even before, they were studied as the uncharacterized inducers of the hybrid dysgenesis phenomenon [50, 51], in which the transmission of some genetic factor by the male but not the female resulted in a sterile progeny. Since then, research on TE in *Drosophila* heavily benefited from the advantages provided by this model, from genetic engineering to sequencing techniques. Not only the molecular mechanisms beyond the hybrid dysgenesis are now much better understood, but the study of this phenomenon also led to major discoveries in TE regulation, such as regulation by small RNAs. In this review, we aimed at giving an overview of the accumulated knowledge on Transposable Elements from molecular aspects to populations genomics in *Drosophila*, comparing the *D. melanogaster* to other *Drosophila* species where relevant.

## TE diversity

### About the classification

The abundance and ubiquity of TEs rapidly brought the necessity of a unified classification system for these sequences. The question of TE classification has been, and continues to be, a subject of debate [11, 52–54], especially the necessity for such system to reflect the phylogeny of TEs. From an evolutionary perspective, a purely phylogenetic classification seems ideal, however this may be hard to achieve. Beyond the polyphyletic nature of TEs, there are several other difficulties. One is that TE phylogeny does not necessarily reflect the organism phylogeny. Another is that the phylogenetic analysis of TE protein sequences may be arduous, because some TEs do not possess any coding sequence, some TEs possess several coding sequences with different phylogenetic signals due to recombination events, and some TEs are present in thousands of copies in the genome. In the sequencing era, when genome annotation is fundamental, Wicker *et al.* (2007) proposed a set of rules to rapidly classify TEs [11]. This widely used classification relies on transposition mechanisms, sequence similarities and structural relationships. In decreasing hierarchical order, we find the following classification levels: class, sometimes subclass, order, superfamily and family (and sometimes subfamily). The highest-level category, *i.e.* class, divides TE sequences into those with or without an RNA transposition intermediate. Next, the order category distinguishes sequences according to the insertion mechanism. Orders are further divided into superfamilies. The superfamily category discriminates sequences on the basis of particular features, for instance protein or non-coding domain structure, presence and length of direct repeats generated on both sides of a TE upon insertion (Target Site Duplication, TSD). The lowest-level category, *i.e.* family, includes sequences with a high rate of identity at the DNA level (at least 80% of identity over at least 80% of their internal or coding domain, or within their terminal repeat regions, or in both). Note that a distinction also exists between autonomous TEs, *i.e.* TEs able to move by themselves, and non-autonomous TEs, *i.e.* TEs relying on other TEs to move, usually because they lack a certain protein.

### Class I TEs: retrotransposons

Class I TEs are also called retrotransposons. They transpose via an RNA intermediate. The RNA intermediate is transcribed from a genomic copy, then reverse-transcribed into DNA by a TE-encoded reverse transcriptase. Each complete replication cycle produces one new copy. Retrotransposons can be divided into five orders: long terminal repeat (*LTR*) *retrotransposons*, Dictyostelium intermediate repeat sequence (DIRS)-like elements, Penelope-like elements (PLEs), long interspersed nuclear elements (LINEs) and short interspersed nuclear elements (SINEs). All of them are present in *Drosophila,* but LTR retrotransposons and LINEs are by far the most abundant [20, 55].

**Fig. 1:**
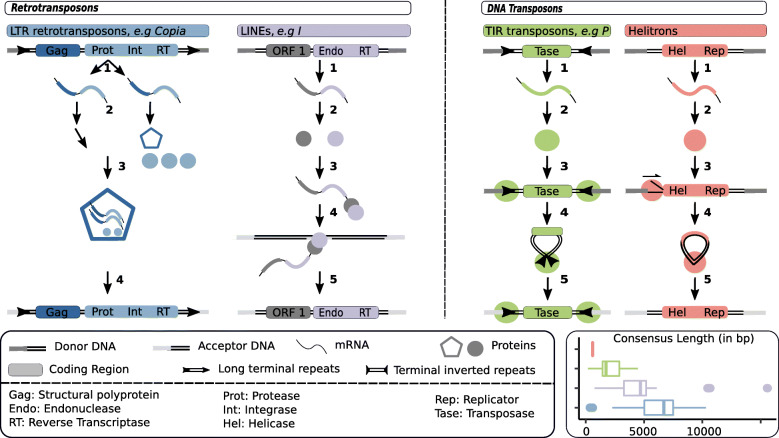
TE structure and transposition mechanisms. LTR retrotransposons: 1. Transcription. 2. Translation of one part of the transcripts. The protease (Prot) cleaves *pol* polyprotein. 3. *gag* proteins assemble around untranslated transcripts, the integrase (Int), reverse transcriptase (RT) and a tRNA. 4. Reverse transcription and integration. LINE retrotransposons: 1. Transcription. 2. Translation. 3. Protein(s) bind to the transcript. 4. A strand of donor DNA is cut, target-primed reverse transcription starts at the exposed 3’ extremity. 5. The TE is integrated. TIR DNA transposons: 1. Transcription. 2. Translation. 3. Two transposases bind to the TIRs. 4. Transposases dimerize and cut TIR extremities forming a free complex. 5. The complex binds to donor DNA and is integrated. Helitron DNA transposons: 1. Transcription. 2. Translation. 3. At the donor site, the plus strand is cut. A replication fork is formed. 4. Replication results in a double stranded transposon circle. 5. Integration. The bottom right panel represents the distribution of the lengths of *D. melanogaster* consensus sequences (RepBase [56]), using the same color code as above.

In *Drosophila*, LTR retrotransposons usually range from 5 to 7 kb (Fig. 1) [11, 57–59]. They owe their names to the direct Long Terminal Repeats (~300-400 bp) flanking them. They typically display two genes: *gag* and *pol*. *gag* encodes the capsid, and *pol* encodes a protease (Prot), an integrase (Int) and a reverse transcriptase (RT) with an RNase domain. After the transcription step, some transcripts will be translated while the others may end up transposed (Fig. 1) (see [60] for more details on transposition mechanisms). The protease of *pol* cleaves Pol into a protease, an integrase and a reverse transcriptase [61]. The Gag protein assemble into a capsid that makes a particle around untranslated transcripts, the integrase, reverse transcriptase and a tRNA [62]. Because the formed ribonucleoprotein (RNP) does not comprise the transcript from which proteins were translated, we typically refer to a trans-preference mechanism of RNP assembly. Using the tRNA as a primer for synthesis, the reverse transcriptase initiates the production of double stranded DNA from the TE transcript [63]. After reverse transcription, the particle falls apart, the integrase recognizes the two ends of the cDNA and inserts them into the host genome. Upon integration, LTR retrotransposons produce a TSD of 4-6 bp [11]. Note that the LTR order is further divided into five superfamilies: *Copia* (*e.g. Copia* and *1731* families), *Gypsy* (*e.g. HMSBEAGLE* and *412* families), *Bel-Pao* (*e.g. BEL, Roo* and *Max* families), *Retrovirus* and *Endogenous RetroViruses (ERV)*. According to Wicker and colleagues classification, *Retroviruses* and *ERVs* also have an envelope gene (*env*). The corresponding protein allows *Retroviruses* to infect other cells. In *Drosophila*, few families have been shown to possess an *env* coding ORF, for example *Idefix*, *Gypsy*, *Tirant* and *ZAM* families [58, 64, 65]. Note that the insect endogenous retroviruses belong to the *Gypsy* superfamily, and that their origin is distinct from that of vertebrate ERVs [66]. Infectious properties have been demonstrated for *Gypsy* and *ZAM* families [67, 68].

LINEs are 3 to 5 kb-long, and generally contain two ORFs (Fig. 1) [11, 59, 69–71]. The first ORF encodes a protein with both RNA binding and nucleic acid chaperone properties [72, 73]. The second ORF encodes a protein that displays two domains: an endonuclease (Endo) and a Reverse Transcriptase [74, 75]. Contrary to LTR retrotransposons, LINEs exhibit a cis-preference mechanism of RNP assembly. After translation, the protein(s) bind to the mRNA molecule from which they originate, and form an RNP in the cytoplasm [76] (see [77] for more details on transposition mechanisms). The ribonucleoprotein particle moves back to the nucleus, and the protein cuts a single strand of the host genome at the point of insertion. The exposed 3’ end allows the initiation of reverse transcription (target-primed reverse transcription). Subsequent events remains unclear, however the following has been proposed. During or after reverse transcription, the second strand of the host genome is cleaved. The newly reverse transcribed single-stranded DNA binds to the generated 3’ extremity, and this extremity acts as a primer for the synthesis of the second strand of DNA. LINEs generate TSDs of various sizes upon insertion. Note that, probably as a consequence of early termination of reverse transcription, transposition may result in creation of 5’ - truncated copies [78].

As mentioned above, besides LTR retrotransposons and LINEs that are abundant in *Drosophila* genomes, Class I comprises three other orders: DIRS, PLEs and SINEs. To our knowledge, DIRS and SINEs have not been found in Drosophila so far [20, 79]. PLEs were initially discovered in *D*. *virilis* and are involved in the hybrid dysgenesis phenomenon (Table 1). These TEs are present at least in the *virilis* group and in *D. willistoni* [89]. PLEs resemble LINEs, in a sense that they encode an endonuclease and a reverse transcriptase. However, they possess terminal repeats that can be in a direct or an inverse orientation.

**Table 1:** Hybrid dysgenesis

In *Drosophila*, some intraspecific crosses were observed to produce sterile females [50, 51, 80–85]. This phenomenon is called hybrid dysgenesis (Ovaries pictures from ref 81). It happens when males possessing a particular TE, hereafter referred as the inducer TE, are crossed with females whose genome is devoid of this TE. On the contrary, the reciprocal cross leads to viable and fertile individuals. The explanation is related to piwi-interacting RNAs (piRNAs), small RNAs repressing TEs with sequence complementarity (see the piRNA section). Because piRNAs are maternally transmitted, in dysgenic crosses the inducer TE insertion is transmitted to the progeny without the piRNAs directed against it [86]. In the reciprocal cross, both the inducer TE insertion and its piRNAs are transmitted, allowing the control of the TE family in the progeny, and the hybrid to be fertile. Hybrid dysgenesis was documented in three systems in *D. melanogaster:* associated with *P-element*, *I-element* or *Hobo* [50, 51, 80]. *P-element* also appears to induce hybrid dysgenesis in *D. simulans* [81]*.* In addition, in *D. virilis,* a hybrid dysgenic cross potentially implying several TEs was reported [82–85]. From a historical perspective, the hybrid dysgenesis phenomenon played an important role not only in the discovery of horizontal transfers of TEs, but also in the study of host defenses against TEs [86–88].	

### Class II TEs: DNA transposons

Class II TEs are DNA transposons. They do not transpose via an RNA intermediate but via a DNA intermediate. There are four orders: terminal inverted repeat (TIR) transposons, Crypton, Helitron and Maverick. TIRs and Helitrons are the most abundant in *Drosophila*.

TIR Transposable Elements are typically ranging from 1.5 to 3 kb in *D. melanogaster*, and are characterized by their TIRs of variable lengths (Fig. 1) [11, 59, 90, 91]. TIRs encode one unique protein called transposase (Tase). The transposition mechanism begins with two transposases recognizing and binding to the TIRs [92]. Transposases dimerize and cleave the ends of TIRs forming a free complex containing the TE [93]. The formed entity binds to the target DNA locus, where the transposon is integrated. The TSD size and the sequences of TIRs are highly variable across the nine known superfamilies [11]. Although the transposition mechanism in itself is not replicative, such TEs can increase their copy numbers in two ways. First, by transposing during chromosomal replication from a position that has already been replicated to a position ahead of the replication fork [94]. Second, they can exploit gap repair following excision to create an extra copy at the donor site [95].

The Helitron order, which is represented by the unique Helitron superfamily, gave rise to rather small TEs in *D. melanogaster* (< 1 kb, Fig. 1) [11, 96, 97]. Helitrons encode one unique protein with both a DNA helicase (Hel) and a replicator (Rep) domain. Because Helitrons were discovered only in 2001, and the lack of active Helitron examples limits experimental work, Helitron transposition mechanisms remain murky. However, using an artificially reconstructed active Helitron, Grabundzija and colleagues provided new insights and suggested the model synthesized hereafter [98]. First, the plus strand, the original donor strand, is nicked at the 5’-extremity of the TE and a replication fork is created. DNA replication results in a reconstituted double stranded donor site and a double stranded TE circle. This step may be repeated several times, producing several TE circles. Moreover, on the TE circles, a second DNA cleavage may occur on the original donor strand, a new replication fork established, and two double stranded transposon circles obtained from one. Finally, the double stranded TE may be integrated at the acceptor site. Note that the small sizes of Helitrons in *D. melanogaster* are explained by their non-autonomous character.

## TE abundance

### The Drosophila melanogaster reference genome

**Fig. 2:**
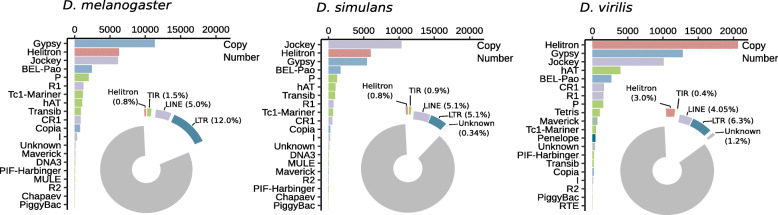
TE contents in *D. melanogaster*, *D. simulans* and *D. virilis* (from left to right). Barplots represent TE copy numbers for the top 20 TE superfamilies. Piecharts illustrate genomic sequence occupancy of each TE order (in percentages of the assemblies). These results were obtained using the *D. melanogaster* reference genome assembly (r6.29), and recently produced long-reads assemblies of *D. simulans* and *D. virilis* [99]. RepeatMasker was used to recover TE fragments and TE genomic sequence occupancy (RepeatMasker v1.332, -nolow, -norna, -species drosophila; Repbase-derived RepeatMasker libraries 20181026 [100],). TE fragments were assembled into TE copies using OneCodeToFindThemAll [101].

To obtain a picture of TE content in *D. melanogaster* genome, we investigated TE copy numbers and TE sequence occupancy in the last release of the reference genome assembly (Fig. 2). We used a combination of RepeatMasker, to identify genomic fragments homologous to a library of *Drosophila* TE consensus sequences available in the RepBase database, and the bioinformatic tool OneCodeToFindThemAll to reconstitute TE copies [56, 100, 101]. As previously reported, *D. melanogaster* genome contains ~20% of TEs [55, 102]. Note that a significant variation exists regarding these estimates [103–105]. These differences are likely to be at least partly explained by the genome assembly, or the part of the genome assembly that is analyzed, or both. For example, the *Drosophila 12 genomes* consortium considered only the best-assembled part of the genome, likely representative of the euchromatic portion of the genome, and found the TE content ranging from 2 % to 8 % (see Population Genomics section for details about TE density in different genomic regions). On the contrary, even if far from reporting the entire sequence of heterochromatic regions, the assembly used in Fig. 2 comprises at least 20 Mb of heterochromatic sequences, *i.e.* ~15% of the 140 Mb assembly [106]. Nevertheless, the relative abundance of the different TE orders is globally conserved across studies and similar to what is represented in Fig. 2 [55, 102, 103, 105]. Retrotransposons, and essentially LTRs and LINEs (respectively 12% and 5% of the genome in our analysis), contribute substantially to *D. melanogaster* TE content. DNA transposons correspond to a smaller proportion of the genome: we found that they represent less than 2%, including 0.9% for Helitrons and 0.7% for TIR elements. This ten-fold difference in terms of genomic sequence occupancy between retrotransposons and DNA transposons is mostly due to the larger size of retrotransposons (Fig. 2). Indeed, in terms of insertion numbers we found 11,657 DNA transposons (6,284 Helitrons and 5,373 TIR elements) and 23,148 retrotransposons (14,540 LTR retrotransposons and 8,608 LINEs) (see also [103] and [101]). For each of the four major orders, one superfamily is often over-represented: *Gypsy* for LTR elements, *Jockey* for LINEs, *P* for TIR elements, *Helitron* for Helitrons. According to our analysis, the different TE orders exhibit different numbers of families: indeed, we found insertions belonging to 721 LTR families, 331 LINE families, 213 TIR families and 63 Helitron families. The mean copy number per family is 26, but large variations exist. The family having the highest number of insertions is DNAREP1_DM, for which we found 1,746 copies. This sequence is annotated as a non-autonomous Helitron [107] (but see [97, 108] concerning classification).

### Interspecific variation

When it comes to TE contents across *Drosophila* species, a direct comparison of studies may be difficult. Indeed, authors are free to choose among a large number of programs and methods dedicated to identifying TEs, which leads to widely different results [105, 109]. For example, using the same TE sequence library but two different tools to annotate the *D. willistoni* genome, the 12 genomes consortium estimated TE content to be either 9 % or 16 %. The library used may also greatly affect results. In the same study, using the same tool, but a *D. melanogaster* TE sequence library or a *de novo* library, the authors found either 12 or 20 % TEs in the *D. ananassae* genome. Overall, in this study seven combinations of library-detection tools were used, leading to a TE content ranging from less than 10 % to up to 30 % in *D. ananassae*. The direct comparison of studies may thus be risky. A further layer of complexity comes from the sequencing technology, which impacts the quality of genome assemblies. Short paired-end read based assemblies lead to underestimation of TE contents compared to Sanger and long read based assemblies [110–112]. For all these reasons, to describe variation of TE contents in the *Drosophila* genus, here we focus on studies directly aiming at comparing TE amounts across species, and we remain cautious when linking them. For illustrative purposes, in addition to the annotation of TE contents in *D. melanogaster*, we estimated TE genomic sequence occupancy and copy numbers in two species: *D. simulans* and *D. virilis* (Fig. 2). We used the exact same methods as for *D. melanogaster,* and we do not expect the TE library to strongly bias the results, as it contains sequences constructed from the three species, which are among the most - studied with regard to TEs [113, 114]. Beyond that, we chose these two species because of their different positions relatively to *D. melanogaster* in the *Drosophila* phylogeny. On one hand, *D. simulans* is a close relative to *D. melanogaster;* they diverged approximately 1.5 Mya. Both species belong to the *melanogaster* subgroup within the *melanogaster* group, itself in the *sophophora* subgenus [14]. On the other hand, *D. melanogaster* and *D. virilis* diverged about 25 Mya and *D. virilis* belongs to a different subgenus, the *drosophila* subgenus.

The first study intending to compare global TE contents across a significant number of *Drosophila* species was performed by the *Drosophila 12 genomes* consortium. This consortium investigated TE genomic sequence occupancy in eight species from the *sophophora* subgenus, mostly from the melanogaster subgroup, and four species from the *drosophila* subgenus. As stated above, the researchers focused on genomic parts likely to be euchromatic, and they used different methods. Using the method giving the lowest estimates, they found a global range of variation going from 1% to 9% of TEs in the genome. The method leading to the highest estimates resulted in genome containing from 3% to 30% of TEs. Invariably, *D. ananassae* was the species with the highest proportion of TEs. The authors chose the most unbiased and conservative method to compare the relative abundance of LTR retrotransposons, LINEs, TIR elements and so-called OTHERs among species. They found that the pattern LTRs>LINEs>TIRs>OTHERs is globally conserved across the phylogeny, with LTR retrotransposons usually constituting more than 50% of the repeatome. The two exceptions are *D. mojavensis* and *D. pseudoobscura.* In *D. mojavensis*, LTR elements represent only 45% of the repeatome, and in *D. pseudoobscura*, LTR retrotransposons and LINEs each contribute to roughly 33% of the repeatome. Our analysis shows a slightly different pattern, with equivalent genomic sequence occupancy for LTR elements and LINEs in *D. simulans*, and more Helitrons than TIR elements in *D. virilis* (Fig. 2). Recently, Hill and colleagues investigated both the proportion of TEs and their number of insertions in the genomes of five species. Four of these species were already in the set analyzed by the *Drosophila 12 genomes* consortium, except for *D. innubila*. The LTRs>LINEs>TIRs>OTHERs pattern for TE genomic proportions was not respected by any of the considered species. The dominant category differed: the most abundant elements are LTR retrotransposons in *D. ananassae,* while they are LINEs in *D. pseudoobscura*, and DNA transposons in *D. innubila*. *D. ananassae* was also the species with the highest TE content, with approximately 35% of TEs in the genome. Considering TE copy numbers, the authors found a total ranging from 2,000 to 14,000 depending on the species. Once again, the difference with the previous results may probably be explained by data/method differences. Relative abundances of the different TE categories were found to differ across genomes. For example, DNA transposons were the most abundant in *D. willistoni,* whereas in *D. ananassae* they were as numerous as LINEs or LTR elements. The study with the largest dataset of species compared in terms of TE content was published by Sessegolo and collaborators [20]. These authors investigated the TE contents of 26 *Drosophila* species. Once again, the LTRs>LINEs>TIRs>OTHERs pattern did not hold for many species. The genomic content of repeats ranged from 4.65% in *D. busckii* to 30.80% in *D. suzukii*. The authors found a significant effect of phylogenetic inertia on TE content, but because of uneven sampling across the phylogeny, it was difficult to extract a pattern for each subgroup, many being represented by only one species. Overall, the data suggest large variations in the abundance of TEs across the *Drosophila* genus.

### Intraspecific variation

At the intraspecific level, genome size, which is correlated to TE abundance in *Drosophila*, is variable within populations of both *D. simulans* and *D. melanogaster*. This suggests that TE contents may change between populations, at least quantitatively [20, 55, 115]. In addition, the discovery of hybrid dysgenesis, *i.e.* the generation of a sterile hybrid by crossing particular parental strains differing by TE families, has highlighted qualitative differences in TE content at the intraspecific level (Table 1) [50, 51, 69]. TE contents in populations were extensively studied by *in situ* hybridization on polytene chromosomes, restricting the results to a few families. Quantitative differences related to the hybrid dysgenesis phenomenon have been observed for *I-Element*, *P-Element* and *Hobo* in *D. melanogaster* [69, 80, 116]. It has been demonstrated that the P-element has recently been acquired by horizontal transfer, likely from *D. willistoni*, and then spread step by step in worldwide populations between 1950 and 1990 [87, 117–119]. The history seems to repeat itself with the current invasion of *D. simulans* by the *P-element* after a horizontal transfer event from *D. melanogaster* [81, 120]. Horizontal transfers of TEs have now been extensively described in eukaryotes [121] and the study of TEs in the genomes of *D. melanogaster*, *D. simulans* and *D. yakuba* suggests that one-third of TE families has originated by recent horizontal transfers between these species [122]. In addition to hybrid dysgenesis, the study of 34 TE families from various populations of *D. simulans* by Vieira and colleagues showed fairly large qualitative differences between populations. Indeed, they found at least 14 families of TEs that were present only in certain populations [123, 124]. Quantitatively, and as an example, a study of the *412* element in *D. simulans* showed a gradient in copy numbers ranging from 1–10 in South Africa to 23 in Europe [125]. Genome size and TE content variations parallel the worldwide colonization of *D. melanogaster* but not that of *D. simulans* [115]. In *D. subobscura*, *Bilbo* and *Gypsy* families show slightly more copies in colonizing than original populations [126]. Similar results were obtained when contrasting copy numbers of *Bilbo* and *Osvaldo* between colonizing and original populations of *D. buzzatii* [127]. In both cases, the study of insertion frequencies suggested that genetic drift associated with a founder effect that accompanied the colonization was responsible for the observed variation of copy numbers. Recently, genomic analyses of European *D. melanogaster* populations from *DrosEU* confirmed that intraspecific variation of TE contents may be substantial, and reveals TE proportions ranging from 16% to 21% of genomes [28].

## TE activity

### Spontaneous rate of transposition

A recent study by Adrion and colleagues [128] provided the first genome-wide estimate of TE movement rate in *D. melanogaster*. These authors used NGS data to compare TE contents across laboratory lines before and after ~150 generations of mutation accumulation. They found that the TE movement rate is slightly lower than the point mutation rate: 2.45 × 10(-9) per site per generation against 2.8 × 10(-9) per site per generation, respectively [129]. The rate of insertions is higher than the rate of deletions: 2.11 × 10(-9) per site per generation against 1.37 × 10(-10) per site per generation, respectively. Considering that there are 270 millions sites in the genome assembly, these numbers correspond to approximately 0.57 insertions and 0.037 deletions per generation. Those estimates were obtained across all TE superfamilies and are consistent with previous reports using *in situ* hybridization to determine transposition events for one or a few families [130–132]. Adrion and colleagues found superfamily-specific insertion and deletion rates to range between 0 and 5.13 × 10(-3) per copy per generation, and between 0 and 1.29 × 10(-4) per generation, respectively. They also found a significant effect of the genetic background, as previously reported [133–135].

### Transposition bursts

Beyond the spontaneous rate of transposition, a significant number of studies have shown that transposition bursts could occur in *Drosophila* (see [136] for a review). A burst is characterized by movement of large numbers of TE sequences through the genome during a short evolutionary time [137]. Although these bursts can happen without any apparent reason, they are commonly associated with stressful conditions such as extreme temperatures, irradiation, chemical exposure, or viral infection [138–142]. For example, Vasil’eva and colleagues showed that gamma radiation could increase the *412* transposition rate up to 5.6 events per genome per generation. Note that the attempts to induce TE mobilization with thermal shocks led to contradictory results in *Drosophila*, potentially due to the differences between tested genetic backgrounds, or tested TEs, or both, but also to methodological considerations (see [136]). Furthermore, although to our knowledge it has not been observed in *Drosophila* so far, stress may also lead to repression of TE activity [143]. Another stress widely studied in *Drosophila* for its effect on transposition is the genomic stress occurring when two somehow divergent genomes are united after hybridization (Table 1). In several biological systems it increases TE activity with potentially dramatic consequences on the phenotype, including sterility [144, 145]. It was observed when crossing individuals from different species, but also when crossing particular strains from the same species which corresponds to the hybrid dysgenesis phenomenon mentioned above [47, 50, 51, 80]. The causes of the TE bursts are not completely elucidated yet. Concerning hybridization, it has been shown that a failure of the host defense against TEs could be at stake (see below and Table 1). Regarding TE activation in response to stressful conditions, it has long been suggested that it could be due to TEs displaying binding sites for stress specific transcription activators, such as transcription factors [146]. In agreement with this idea, the temperature responding M*ariner* and C*opia* elements were shown to display sequences homologous to the promoter of heat shock proteins [147, 148]. More recently, a transcriptomic study demonstrated that temperature dependent TE expression is TE family specific and dependent on the genetic background. The authors proposed that TE transcription is indeed regulated by an interaction between TE family-specific regulatory sequences and host *trans*-acting factors [149]. Note, however, that this study was done on a range of temperatures that are not necessarily stressful (13–29°C). It is also important to consider that all the reports mentioned above concern laboratory experiments in conditions that are potentially unlikely *in natura*. The mechanisms at play in natural populations still remain poorly understood. One study demonstrated a burst of transposition for *DINE-1* in *D. yakuba* [150], and its causes are still unknown. In *D. simulans,* the copy numbers of the *412* element increase with latitude following the minimum temperature, and in *D. melanogaster*, significant correlations were found between TE abundance and different geographical and environmental variables for four families [125, 151]. However, in both cases, a possible confounding effect of demographic history cannot be excluded. Only one study established a direct link between TE activity and a geo-climatic variable: in *D. simulans,* the *Mariner* element somatic activity varies along a latitudinal cline between tropical Africa and Europe [152].

### Interspecific variation

**Fig. 3:**
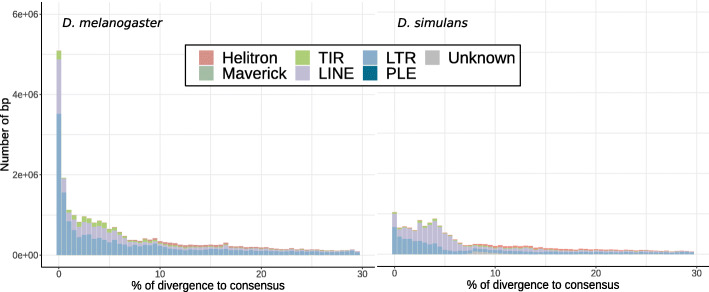
TE landscapes in *D. melanogaster* and *D. simulans*. For each TE fragment the divergence to consensus was estimated. For each TE order the total amount of DNA (in bp) is shown as a function of the percentage of divergence. The percentage of divergence to the consensus sequences is a proxy for age: old TEs have accumulated mutations, young TEs are similar to consensus sequences. RepeatMasker was used to recover TE fragments in genomic assemblies (same method as Figure 2 [100],). Percentages of divergence to consensus were evaluated from RepeatMasker output .align file using A. Kapusta script [154]

So far, few studies tried to compare TE activity across *Drosophila* species. In 2011, Lerat and colleagues compared the TE contents of four *Drosophila species* from the *melanogaster* subgroup: *D. melanogaster*, *D. simulans*, *D. sechellia* and *D. yakuba* [153]. They found that *D. simulans, D. sechellia* and *D. yakuba* genomes contained a large fraction of degraded copies compared to *D. melanogaster*. The authors suggested a recent TE activity in *D. melanogaster*, compared to the three other species. This can partially be observed when comparing the so-called TE landscapes of *D. melanogaster* and *D. simulans* (Fig. 3). These landscapes constitute an easy way to visualize TE activity through time. The X axis corresponds to the divergence of the TE sequences from the consensus, and it can be seen as a proxy of the time passed since the last wave of transposition. In Fig. 3, we can see a recent peak of activity of LTR elements, especially in *D. melanogaster*. In *D. simulans*, the peak of activity is also recent but much smaller. Another study was aimed at comparing TE activity between *D. melanogaster* and *D. simulans* using NGS population data [155]. Based on TE insertion frequency data, the authors determined that more than 58 families are probably highly active in both species. Half of the TE families show evidence of variation of activity through time, and are not the same depending on the species. Finally, they found that retrotransposons were the most active TEs in *D. melanogaster,* while DNA transposons were the most active TEs in *D. simulans*. A recent study compared TE frequencies in five distant points of the *Drosophila* phylogeny [55]. These species shared a common ancestor around 30 Mya [14]. The authors found evidence that an excess of low frequency insertions is prevailing in the phylogeny and is observed for most TE families. This suggests that an active repeatome is frequent, at least in the *Drosophila* genus.

## Impacts of TEs

### On the genome

TEs play an important role in the structural evolution of genomes through the generation of various types of mutations: chromosomal rearrangements, gene disruption and changes in gene expression. The simplest mechanism by which TEs can cause chromosomal rearrangements is through participation in an ectopic recombination event [156]. Ectopic recombination corresponds to recombination between more-or-less identical sequences inserted at different locations in the genome, such as TEs [157]. Depending on their relative positions and orientations, their recombination can result in different kinds of chromosomal rearrangements: duplication, deletion, inversion, or translocation. TEs were associated with chromosomal rearrangements *in natura* in various species of *Drosophila*, and mainly with inversions [158–161]. In several cases, ectopic recombination was identified as the cause of these rearrangements [159, 160]. When they insert into genes or their regulatory sequences, TEs can disrupt gene function. A perfect example is the use of the *P-element* in the Berkeley *Drosophila* Genome Project [162–164]. The Berkeley *Drosophila* Genome Project aimed at disrupting each *D. melanogaster* gene using the *P-element* in order to decipher gene functions. More than 5,000 genes were disrupted in that way. TEs can affect gene expression in two principal ways. First, they may bring regulatory sequences (see [165] for a review). For example, *Bari-Jheh* adds extra antioxidant response elements upstream of the *Jheh1* and *Jheh2* genes and is associated with upregulation of *Jheh1* and *Jheh2* [166]. Second, the spread of repressive epigenetic marks targeting TEs can reduce the expression of nearby genes (see below, host defenses against TEs), as it was also demonstrated in the *Jheh* cluster [167]. Lee and Karpen demonstrated recently that the spread of repressive epigenetic marks to nearby DNA occurs for more than half of euchromatic TEs, and can extend up to 20 kb [12]. This effect is TE dependent, copy number dependent, but also species dependent, with stronger epigenetic effect in *D. simulans* compared to *D. melanogaster*.

### On the individual

While some of the aforementioned genomic changes might remain phenotypicaly silent, others may have dramatic repercussions at the individual level. TEs are responsible for up to 80% of the phenotypic spontaneous mutations observed in *D. melanogaster* [168] and many observations suggest deleterious effects of TEs in *Drosophila*. Five to 10 % insertions of active *P-elements* are estimated to cause recessive lethal mutations in *D. melanogaster* [169]. In *D. simulans,* somatic transposition of *Mariner* decreases lifespan [170]. In 2004, a study used two *D. melanogaster* lines with the same genetic background, but different TE copy numbers, to evaluate the impact of TE number on fitness. The authors found differences in fitness and egg hatchability between the two lines, the line with more TEs performing worse than the other. Both homozygous and heterozygous TE insertions were shown to have deleterious effects on fitness and its components [134]. Overall, TE insertions are expected to be generally neutral or deleterious to the host genome [171]. Considering that adaptive mutations are supposed to quickly reach fixation in populations, the low numbers of fixed insertions in *D. melanogaster* and *D. simulans* support this theory. In 2006, Burt and Trivers calculated the number of insertions since the divergence between the two species and concluded that, given both genome size and number of fixed insertions, the occurrence and fixation of a beneficial insertion is a really rare event [156]. However, they also underscored the difficulty to detect fixed insertions using *in situ* hybridization, and suggested it would have been interesting to estimate the rate of fixation from sequencing data. In 2015, using population sequencing data, Kofler and colleagues estimated the number of fixed insertions in *D. melanogaster* since its divergence from *D. simulans* to be approximately 200 [155]. Considering a 1.4 Mya divergence [14], we computed a fixation rate of 1.4 fixed insertions every 10,000 years, *i.e.* maximum 1.4 beneficial fixed insertions every 10,000 years. If we update the Burt and Trivers calculation and compare the number of fixed insertions to the total number of insertions over this period: Population size × Insertion rate per genome per generation × Divergence time between *D. melanogaster* and *D. simulans* × Number of generations per year = 10 (6) × 0.57 × 1.4 × 10 (6) × 24 = 1.9 × 10 (13) insertions, that is to say 200/(1.9 × 10 (13)) = 1.0 × 10(-11) insertions reaching fixation. Finally, we estimated maximum 1.4 beneficial fixed insertions every 10,000 years, or maximum 1 out of 1e11 insertions, being beneficial and fixed. These numbers are upper bounds because all fixed insertions are unlikely to be beneficial. Indeed, most of the fixed insertions are present in regions where the effect of selection is weak, and are essentially old. Therefore, they are more likely to have reached fixation slowly by drift than quickly by positive selection [172, 173]. So far, 21 fixed insertions have been identified within or near genomic regions showing low Tajima’s D values, and 12 fixed insertions are relatively young. Considering the above, one could expect to find very few putatively adaptive insertions among unfixed insertions. Surprisingly, there are at least 57 of such insertions in the reference genome [173], suggesting a high rate of TE mediated adaptation recently or even ongoing. The discrepancy between the number of candidates for recent adaptation and the fixation rate was discussed considering the three following points: 1. The migration of *D. melanogaster* out of Africa may have caused a significant augmentation of the adaptation rate. 2. TE derived adaptations might be ephemeral. 3. Adaptive TE sequences may evolve quicker than neutral insertions, resulting in an underestimation of the number of fixed insertions [174]. One may also add that the TE mutation rate has potentially increased recently [175]. It is worth noting that few insertions were clearly associated with an adaptive phenotype so far [166, 176–178]. Interestingly, candidate adaptive insertions are often close to, or within genes associated with stress response, behavior and development. Moreover, two of the historical examples of adaptation associated with TEs correspond to two different insertions in the same gene implicated in the response to oxidative stress, *cyp6g1,* in two different species: *D. melanogaster* and *D. simulans* [176, 178, 179].

### The case of telomeric elements

A few TEs appear to have evolved a new function in *Drosophila* genomes. Because of the DNA replication mechanism, a *Drosophila* chromosome end loses 70-80 bp each generation [180]. This gradual reduction of chromosome ends is threatening internal regions containing essential genes and may contribute to ageing [181]. Organisms have evolved different mechanisms that protect their chromosomes. Usually in eukaryotic genomes a ribonucleoprotein enzyme, the telomerase, mediates the RNA dependent synthesis of tandemly repeated simple sequences at chromosome ends [182]. In *D. melanogaster*, the three families, *HeT-A*, *TART* and *TAHRE,* transpose to chromosome extremities, and protect them from shortening [180, 183–186]. Many phylogenetically distinct telomeric retrotransposons have been found in more distant species [187]. All these telomeric elements belong to a single monophyletic clade inside the *Jockey* superfamily. The telomeric element phylogeny and species phylogeny are congruent, suggesting vertical transmission from a common ancestor and a conserved host-element relationship [187]. Furthermore, the clade presents evidence of specialization to transpose at chromosome ends [188]. Because of this, the relationship between TEs and their host in this case was referred to as genomic “symbiosis” [188]. However, Saint-Leandre and colleagues investigated more species of the *melanogaster* group [189]. They suggest that these *Jockey* telomeric elements may have evolved to selfishly over-replicate. In agreement with this hypothesis, they found recurrent gains, losses, and replacements of *Jockey* telomeric elements. Moreover, in *D. biarmipes*, the telomere-specialized elements have disappeared completely.

## Host defenses

Because of the above-mentioned deleterious effect of TE insertions, several mechanisms of TE control have evolved. Among these, epigenetic modifications play an important role [190]. For example, in mammals and plants, TE insertions are usually associated with DNA methylation and histone modifications. Both are related to repressive chromatin states. In *Drosophila*, DNA methylation has been shown to be almost completely absent, and small RNAs are central to TE regulation [191, 192]. They may also trigger histone tail modifications and chromatin conformation modifications. There are two small RNA pathways controlling TEs in *Drosophila*: the piRNA and the siRNA pathways. Our purpose here is to give a brief overview of these pathways and their role in shaping TE dynamics. In particular, we refer the reader to [193, 194] for comprehensive reviews on the mechanistic aspects of the piRNA pathway.

### The piRNA pathway

The piRNA pathway produces small, single stranded RNAs that were first called rasiRNAs (repeat associated small interfering RNAs); however, contrary to regular small interfering RNAs, they are 23-30 nt long, and are associated with the Piwi-subfamily Argonaute proteins, which led to their new designation as piRNAs (piwi-interacting RNAs). These piRNAs silence TEs in germ cells, where maintaining the integrity of the genome is of primary importance, as new mutations are passed on to future generations. This pathway is also active in the ovarian somatic follicle cells, which support oogenesis. It prevents endogenous retroviruses, such as *Gypsy,* from infecting the adjacent oocyte [195]. Research studies in *Drosophila* were seminal in the piRNA field. Much of what we know today was discovered using this model. In fact, piRNAs were identified for the first time in 2001 in fly testis [196]. They were found to silence *Stellate,* a gene involved in male sterility. Some of them were even found to be homologous to TEs and assumed to be involved in transposon regulation. Moreover, a long-term study of the *Gypsy* family activity led to the discovery of *flamenco*, a non protein-coding locus producing piRNAs, which was subsequently shown to be involved in the control of other TE families, essentially LTR retrotransposons [197, 198] (Table 2).

**Table 2:** the *flamenco* story

Much of what we know today on the piRNA pathway was discovered using the *Drosophila* model. Especially, a considerable effort started 40 years ago in *D. melanogaster* led to the early discovery of a gene producing piRNAs and silencing *Gypsy* in a piwi dependent manner (see [199] for a review). This gene named *flamenco* was the first piRNA cluster identified. In the 1980s, the *ovoD* dominant mutation was identified in *D. melanogaster* and associated with female sterility [200]. Interestingly, crosses between *ovoD* males and females from a particular strain led to the reversion of the phenotype and the recovery of fertility of the daughters, in addition to numerous mutations at other loci. Further work revealed that the particular strain used for the mothers actually displayed high copy numbers of uncontrolled *Gypsy*, whose transposition into *ovo* led to a null allele and reversion of sterility [201]. And then, it was demonstrated that the locus controlling *Gypsy* activity was the *flamenco* locus, located on the X chromosome and containing a lot of TE sequences [202, 203]. In 2004, Sarot and collaborators found out that *Gypsy* transposition was sensitive to a mutation in *piwi*, a gene known to affect RNA-mediated silencing [204]. They also demonstrated that small RNAs homologous to *Gypsy* where present in silenced tissues. For the first time a gene producing small RNAs was associated with TE silencing, and Piwi was implicated in this process. Despite the subsequent discovery of many other piRNA clusters in *D. melanogaster*, flamenco is still widely studied as a model and produces most of the piRNAs in ovarian somatic cells [197, 205–207].	

**Fig. 4:**
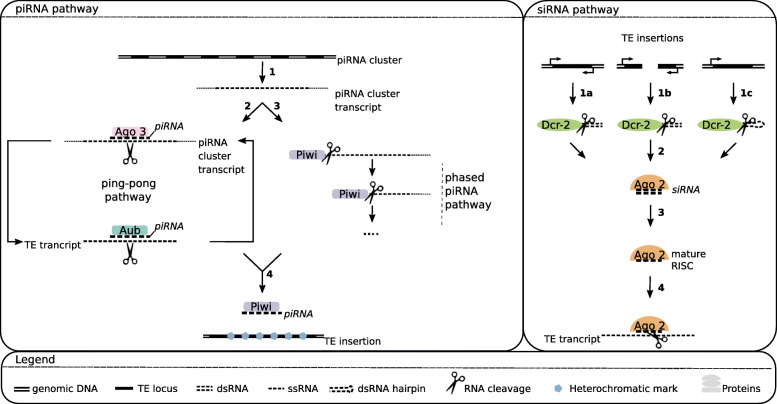
small RNA pathways controlling TEs. piRNA pathway: 1. RNA PolII transcribes a genomic piRNA cluster into a long single stranded RNA. 2. The transcript thus formed enters the ping-pong pathway, which is ensured by Aub and Ago3, generates sense and antisense piRNAs, and ensure post-transcriptional silencing by transcript slicing. 3. Piwi directs the cleavage of the piRNA cluster transcript and generates a piRNA. This step may be repeated. 4. Transcriptional silencing: in the nucleus, a piRNA guides Piwi and promotes H3K9 methylation of TE DNA sequences. siRNA pathway: 1. Generation of a long dsRNA by: a. bi-directional transcription of a unique TE locus, b. interaction of two complementary transcripts from distinct TE loci. c. hairpin formation, due for example to inverted repeats binding 2. Dcr-2 processes long dsRNAs into siRNAs, which are loaded on Ago2. 3. The passenger strand of the siRNA is sliced by Ago2, only the guide strand remains. 4. The RISC binds to a TE transcript with sequence complementarity to the guide strand and Ago2 cleaves it.

piRNAs originate from discrete genomic loci called piRNAs clusters. These loci contain mainly defective TEs and are transcribed into long piRNA precursors (Fig. 4 [202]). Approximately 150 clusters have been identified in the genome of *D. melanogaster*, representing 3.5% of the assembled genome [208]. The vast majority of them appear to be heterochromatic. The size of piRNA clusters varies substantially, with the largest being 240 kb. Overall, the largest 15 clusters produce a large proportion of the total amount of piRNAs: 70% of the piRNAs uniquely mapped to the genome originate from these clusters.

The beginning of piRNA biogenesis is similar in germline and somatic cells (see [193, 194] for detailed reviews). PiRNA cluster transcription is ensured by RNA Pol II and leads to a single stranded long RNA (Fig. 4). Then, piRNA cluster transcripts may enter either the ping-pong pathway or the phased piRNA pathway [208–213]. The ping-pong pathway occurs in germline cells. In this case, guided by a sense piRNA, Argonaute3 (Ago3) binds to a complementary piRNA cluster transcript and cleaves it. Then, Aubergine (Aub) attaches to the newly formed 5’ extremity, slices the transcript and forms an antisense piRNA. Finally, guided by an antisense piRNA, Aub operates a cut in a TE transcript, Ago3 recognizes the resulting 5’ extremity, cleaves the transcript and forms a sense piRNA. This is the ping-pong pathway or ping-pong loop. The phased piRNA pathway is not specific to germline cells and may also occur in ovarian somatic follicle cells. Piwi is loaded at the 5’ extremity of the piRNA precursor and Zucchini (Zuc) performs cleavage, generating the piRNA. Piwi is then loaded again at the 5’ extremity of the precursor piRNA, and the process is repeated in a step-by-step cleavage generating multiple piRNAs. Note that, for clarity, piRNA maturation steps such as trimming are not mentioned here.

After synthesis, piRNAs mediate silencing both at the transcriptional and post-transcriptional levels [214]. The post-transcriptional silencing occurs in the cytoplasm of germline cells only, and corresponds to the ping-pong pathway (Fig. 4). At the transcriptional level, a piRNA guides the Piwi protein to a TE insertion, probably due to sequence complementarity with nascent TE transcripts, and mediates local heterochromatin formation by addition of the repressive mark H3K9me3 to histone tails [215–221]. Note that, despite the fact that as early as 2001 piRNAs were detected in testes, so far most of the work on TE regulation by piRNAs has been done on ovaries [196]. Regulation in testes seems to be quite similar to what happens in female germline, with both ping-pong and phased piRNA pathways being active [222–224]. However, contrary to ovaries, the data suggest an Ago-3 independent amplification loop in spermatogenesis.

### The siRNA pathway

In addition to piRNAs, sequencing of small RNAs revealed the existence of another class of interfering RNAs targeting TEs: endogenous small interfering RNAs, or endo-siRNAs [225–227]. These small RNAs are present in both somatic and germline cells. endo-siRNA precursors are double strand RNAs (dsRNAs). These precursors may be produced through three distinct mechanisms (Fig. 4) [228]. 1. Transcription of the same genomic region in both sense and antisense directions (convergent transcription), then base pairing of the overlapping region between sense and antisense transcripts. 2. Transcription of complementary sense and antisense transcripts from different genomic regions and base pairing. 3. Base pairing of inverted repetitive elements of one transcript to form a hairpin RNA. The resulting long dsRNA is loaded on Dicer-2 (Dcr-2) and its cofactor Loquacious-PD (Loqs-PD) and then processed into 21 nt small double stranded RNAs. They are then loaded on the *RNA-induced silencing complex (*RISC) including the Ago2 protein. One strand is held and guides the complex to target transcripts that are then cleaved by the RNase domain of Ago2.

### Evolution

Several studies demonstrated rapid evolution of anti-TE RNAi genes in *Drosophila* [47, 229–232]. Indeed, these genes often present signatures of recurrent positive selection. By analogy to the signatures of positive selection observed for genes involved in host-parasite interactions, the rapid evolution of anti-TE RNAi genes is often interpreted as a consequence of an arms race occurring between TEs and TE immunity effectors. Focusing on the piRNA pathway, Blumenstiel and colleagues propose that selection for sensitivity to TE content but also selection for specificity to TE content may drive the rapid evolution of host defense mechanisms [233]. More precisely, concerning the specificity aspect, the authors propose that a too efficient piRNA pathway may induce a too efficient silencing of TE copies that could spread to neighboring genes, which would constitute a cost. They designated this form of off-target gene silencing as “genomic autoimmunity”, an analogous to classic forms of autoimmunity which are caused by an immune response that incorrectly targets self. Despite the rapid evolution of anti-TE RNAi genes in *Drosophila*, suggesting that host defense mechanisms may vary a lot across the genus, most of the literature on this subject concerns *D. melanogaster*. A recent study of 20 arthopod species suggests that somatic piRNAs were probably produced in the ancestral arthropod more than 500 Mya and demonstrated that, in contrast to *D. melanogaster*, *D. virilis* presents somatic piRNAs [234]. This suggests a loss of the piRNA pathway in the soma of *D. melanogaster*.

## Population genomics

The *Drosophila* model has been of outstanding importance in the field of population genomics of TEs. The ease to get and maintain wild type strains was obviously a key factor, but so was the development of the *in situ* hybridization method on *Drosophila* polytene chromosomes more than 40 years ago [235, 236]. *In situ* hybridization allows to detect and localize genomic DNA sequences using a labeled sequence (probe) homologous to the targeted sequence. The giant polytene chromosomes are found only in some species and tissues, and offer to the researcher a high degree of resolution [237, 238]. Using TE probes on salivary gland polytene chromosomes of *Drosophila* third instar larvae, researchers were able to detect and localize TE insertions in individuals and thus to accurately estimate TE insertion frequencies in natural populations [239–241].

### About the nature of selection acting on TEs

The first *in situ* hybridization studies evaluating TE insertion frequencies in natural populations of *D. melanogaster* demonstrated a predominance of insertions segregating at low frequencies [239–241]. This result obtained for specific families was later confirmed at a broader scale. Population sequencing data showed that, in *D. melanogaster* and *D. simulans*, more than 80% of TE copies have insertion frequencies lower than 0.2 [155]. This observation is often interpreted as the result of purifying selection acting on TEs. So far, three main hypotheses have been formulated concerning the nature of selection against TEs: 1) the gene-disruption hypothesis [3, 242], 2) the ectopic recombination hypothesis [243, 244], 3) the deleterious TE-product expression hypothesis [245].

The gene disruption hypothesis assumes that insertions inside genes or regulatory regions are under strong purifying selection because of their negative effect on the host fitness [242]. A large amount of work supports this hypothesis, demonstrating a depletion of TE insertions in exons and untranslated regions [172, 246–248]. Moreover, Lee and Karpen demonstrated that repressive histone marks affecting euchromatic TEs can spread up to 20 kb both in *D. melanogaster* and *D. simulans*, and that this phenomenon is associated with selection against TEs [12]. Therefore, we may extend this hypothesis beyond insertions inside genes or regulatory regions to include insertions close to genes.

The ectopic recombination hypothesis states that purifying selection acts against chromosomal rearrangements resulting from recombination events between TE sequences showing sequence identity and located at distinct loci [243, 244]. According to this hypothesis, TE size, TE family copy number, and meiotic recombination rate, expected to be positively correlated with ectopic recombination rate, should be associated with the strength of purifying selection [137]. First, since long insertions provide longer targets for recombination, one can indeed expect a stronger effect of purifying selection against long TEs in the ectopic recombination hypothesis. The negative correlation between TE size and population frequencies suggests that it is actually the case [172, 249]. Second, because ectopic recombination is more likely to occur when TEs are heterozygous, ectopic recombination should happen more frequently for TE families with a high copy number of polymorphic TEs. Therefore, the negative correlation between TE insertion frequencies and copy numbers also supports the ectopic recombination hypothesis [172, 249]. Finally, because ectopic recombination is intrinsically related to the local recombination rate, the fact that low-recombining regions are highly enriched in TEs, and that a negative correlation exists between insertion frequencies and recombination rate [172, 246, 249, 250], constitute one more argument in favor of the ectopic recombination hypothesis. However, this last point may be explained by the Hill-Robertson effect, or the lower density of genes in low-recombining regions, or both. The Hill-Robertson effect corresponds to a reduction in the efficiency of selection on a locus due to selection on related loci. If slightly deleterious insertions are close to adaptive mutations, they will be less efficiently removed in low-recombining regions than in high-recombining regions. The lower density of genes in low-recombining regions may explain the higher TE density in these regions because one may expect that TE insertions are strongly counter-selected close to genes (gene disruption hypothesis). However, one paradox exists when considering the ectopic recombination hypothesis. Indeed, considering the higher rate of recombination on the X chromosome, and the ectopic recombination hypothesis, TE density should be lower on the X chromosome [251]. However, recent studies of *D. melanogaster* natural populations show different results. TE density was found to be either higher on the X chromosome [246], or similar between the X chromosome and autosomes when taking into account differences in the amount of low recombining regions [172]. A higher transposition rate in the X chromosome relatively to autosomes has been proposed as a plausible explanation to the observed paradox [137]. Mutation accumulation data recently showed such tendency with a 1.86 fold change for insertion rate on the X chromosome relatively to autosomes [128].

One last hypothesis remains concerning the nature of the purifying selection affecting TEs: the deleterious TE-product expression hypothesis [245]. Under this model, transcription and translation of TEs may be resource consuming for the host and TE proteins could disrupt cellular processes. According to this hypothesis, and assuming that full length TEs are more transcribed than nearly complete copies, one may expect complete copies to be under more intense purifying selection than nearly complete copies. However, Petrov and colleagues did not find such effect investigating TE frequencies genome wide [249].

### Models of TE dynamics

So far, two main models have been formulated to conceptualize TE dynamics in *Drosophila* populations. The historical model is the transposition-selection balance model: it assumes that TE abundance is regulated by a balance between transposition and selection against TEs [3, 252]. According to this model, insertions with low frequency in populations are expected to be mainly insertions subjected to strong purifying selection. However, because transposition rates are not constant over time, another model has been proposed: the transposition burst model [175]. This model proposes that TE dynamics in populations is explained by transposition bursts. Under this hypothesis, a large proportion of low frequency insertions may result from recent TE activity rather than strong selection against TEs. Data, especially on TE genomic distribution (see above), suggest a preeminent role of purifying selection in TE dynamics, and thus support the transposition-selection balance model. Furthermore, an excess of rare TEs compared to the standard neutral model is found, as expected if selection acts against TEs [246]. However, confronting population data with simulation, Kofler and colleagues showed that both in *D. melanogaster* and *D. simulans*, 50% of families have temporally heterogeneous transposition rates and that a correlation exists between insertion frequencies and their age [155, 172]. So far, it is clear that both purifying selection and variation in transposition rate act on TE population dynamics. Until now, TE regulation has been poorly integrated in the models of TE dynamics. In 2010, Lu and colleagues incorporated piRNAs in a population genetics framework [253]. They used simulations to investigate the dynamics of TEs. They focused on retrotransposons, studying the retrotransposons that are targeted by piRNAs but also the retrotransposons generating piRNAs. The results indicate that: piRNAs may reduce TE fitness cost; TEs generating piRNAs may easily reach fixation because they confer a selective advantage; and TEs targeted by piRNAs may also reach fixation because host defenses reduce their deleterious effect. In 2013, the observation that a TE insertion inside a piRNA cluster was able to silence the corresponding TE family led to the formulation of the trap model [197]**.** In this model, after invasion of a host genome, a TE family proliferates until it is trapped, *i.e.* one insertion occurs into a piRNA cluster, then the subsequent production of piRNAs silences the invading family. This model was validated and enriched with populational considerations by Kofler and colleagues [88]. Monitoring the *P-Element* invasion, in connection with the piRNA pathway, in experimentally evolving populations of *D. simulans*, they suggested the following three-step model for a TE invasion: 1) TE copies colonize the genome, 2) the first TE insertions in piRNA clusters occur but are not yet sufficient to stop TE proliferation and 3) the TE family is inactivated by the fixation of an insertion within a piRNA cluster. Using simulated data, they were able to demonstrate that this “trap model” accurately describes TE abundance in *D. melanogaster* germline. They also showed that the suppression of TE activity by segregating cluster insertions is reversible. Importantly, they demonstrated that transposition rates and population sizes affected mostly the duration of the invasion steps but not the amounts of accumulating TEs. In fact, the major factor capable of affecting the number of accumulating TEs was the piRNA cluster size.

## Conclusions

In today's biology research, increasing weight is given to the study of non-model species. This is clearly justified by the diversity of the living world, and even more so for the study of genetic elements as diverse and dynamic as TEs. However, we should not overlook model organisms, because the vast amount of techniques, data collected and knowledge will help us develop and test new hypotheses. Furthermore, the dissection of conserved pathways in these organisms, such as the piRNA pathway, should provide results valid for a broad range of species. Despite the fact that *Drosophila* is an old biological model, it still presents many opportunities for TE research. In general, studies of TEs could benefit from unified approaches to identifying and quantifying TEs. As we demonstrated above, the ultimate model *D. melanogaster* appears slightly different from its sister species regarding TEs —maybe related to the fact that it ended up as the ultimate model species— however, it is clear that the research community greatly benefits from comparative genomics in the *Drosophila* genus, and a great deal of work remains to be done in *Drosophila* and the species in the group in order to do proper comparative genomics. It is clear that the development of long-read technologies will greatly facilitate this work. Another challenge is to understand the activity of TEs and how, *in natura*, this activity is triggered and controlled. Once again, *Drosophila* is a model of excellence with the possibility of doing experimental evolution with a follow-up of TE dynamics. At the same time, this will allow a better understanding of the fine regulation systems of TE activity. Finally, it seems to us that one of the most exciting challenges is to understand the true impact of TEs in adaptive processes, even more so now, with all the gross changes in our environment. Experimental evolution, with different species and different environmental factors, are a real opportunity to move forward in this field.

## Data Availability

The datasets analyzed during the current study are available in the following repositories: https://github.com/danrdanny/Drosophila15GenomesProject/raw/master/assembledGenomes/ [99], ftp://ftp.flybase.net/genomes/Drosophila_melanogaster/dmel_r6.29_FB2019_04/

## Abbreviations

Ago2: Argonaute2
Ago3: Argonaute3
Aub: Aubergine
Dcr-2: Dicer-2
DIRS: Dictyostelium Intermediate Repeat Sequence
Endo: Endonuclease
Env: Envelope gene
ERV: Endogenous RetroViruses
Int: Integrase
Hel: DNA helicase
LINE: Long Interspersed Nuclear Element
Loqs-PD: Loquacious-PD
LTR: Long Terminal Repeat
piRNA: piwi-interacting RNA
PLE: Penelope-Like Element
Prot: Protease
rasiRNA: repeat associated small interfering RNA
Rep: Replicator
RISC: *RNA-induced silencing complex*
*RNP*: *RiboNucleoProtein*
RT: Reverse Transcriptase
Tase: Transposase
TSD: Target Site Duplication
TE: Transposable Element
TIR: Terminal Inverted Repeat
SINE: Short Interspersed Nuclear Element
siRNA: small interfering RNA
Zuc: Zucchini
